# Impaired Spermatogenesis and gr/gr Deletions Related to Y Chromosome Haplogroups in Korean Men

**DOI:** 10.1371/journal.pone.0043550

**Published:** 2012-08-23

**Authors:** Jin Choi, Seung-Hun Song, Chong Won Bak, Se Ra Sung, Tae Ki Yoon, Dong Ryul Lee, Sung Han Shim

**Affiliations:** 1 Genetics Laboratory, Fertility Center of CHA Gangnam Medical Center, Seoul, South Korea; 2 Department of Urology, Fertility Center of CHA Gangnam Medical Center, CHA University, Seoul, South Korea; 3 Department of Obstetrics and Gynecology, Fertility Center of CHA Gangnam Medical Center, CHA University, Seoul, South Korea; 4 Department of Biomedical Science, CHA University, Seoul, South Korea; Kunming Institute of Zoology, Chinese Academy of Sciences, China

## Abstract

Microdeletion of the Azoospermia Factor (*AZF*) regions in Y chromosome is a well-known genetic cause of male infertility resulting from spermatogenetic impairment. However, the partial deletions of *AZFc* region related to spermatogenetic impairment are controversial. In this study, we characterized partial deletion of *AZFc* region in Korean patients with spermatogenetic impairment and assessed whether the *DAZ* and *CDY1* contributes to the phenotype in patients with gr/gr deletions. Total of 377 patients with azoo-/oligozoospermia and 217controls were analyzed using multiplex polymerase chain reaction (PCR), analysis of *DAZ-CDY*1 sequence family variants (SFVs), and quantitative fluorescent (QF)-PCR. Of the 377 men with impaired spermatogenesis, 59 cases (15.6%) had partial *AZFc* deletions, including 32 gr/gr (8.5%), 22 b2/b3 (5.8%), four b1/b3 (1.1%) and one b3/b4 (0.3%) deletion. In comparison, 14 of 217 normozoospermic controls (6.5%) had partial *AZFc* deletions, including five gr/gr (2.3%) and nine b2/b3 (4.1%) deletions. The frequency of gr/gr deletions was significantly higher in the azoo-/oligozoospermic group than in the normozoospermic control group (*p* = 0.003; OR = 3.933; 95% CI = 1.509–10.250). Concerning Y haplogroup, we observed no significant differences in the frequency of gr/gr deletions between the case and the control groups in the YAP+ lineages, while gr/gr deletion were significantly higher in azoo-/oligozoospermia than normozoospermia in the YAP− lineage (*p* = 0.004; OR = 6.341; 95% CI = 1.472–27.312). Our data suggested that gr/gr deletion is associated with impaired spermatogenesis in Koreans with YAP− lineage, regardless of the gr/gr subtypes.

## Introduction

The azoospermia factor (*AZF*) locus has been mapped to the long arm of the human Y chromosome that is associated with spermatogenetic failure [1], [2]. Four recurrent microdeletions of the *AZF* region related to azoospermia or oligozoospermia have been identified to date: *AZF*a, P5/proximal-P1 (*AZF*b), P5/distal-P1 and *AZF*c (b2/b4) deletions [2], [3]. In addition to these deletions, several partial *AZF*c deletions (gr/gr, b2/b3, b1/b3 and b3/b4), inversion, and duplications resulting from non-allelic homologous recombination have been reported [4]–[11]. Among them, the most clinically relevant type is gr/gr deletion including two copies of the *DAZ* gene and one copy of *CDY*1 gene. *DAZ* and *CDY*1 have been known to be the most important candidates related to spermatogenesis in the *AZF*c region [12]–[14]. Recently, the gr/gr deletion has been newly defined as ‘gr/gr deletion rearrangements’ and was divided into five rearrangement types, simple gr/gr deletion, gr/gr deletion-b2/b4 duplication, gr/gr deletion-b2/b4 multiple duplication, gr/gr deletion-*CDY*1 and *DAZ* amplification [6], [15]. In addition, several other studies showed the relationship between gr/gr deletion subtypes and spermatogenetic impairment but the results were different among populations. In some populations, the deletion showed a significant risk factor for spermatogenetic failure but not in others [4], [7], [14], [16]–[22].

Therefore, this study was designed to characterize the partial *AZF*c deletion patterns and their clinical consequences in Korean population. So, we carried out *AZF*c-STS analysis, gene copy analysis and gene dosage analysis and Y- haplogroup analysis as well.

## Materials and Methods

### Ethics Statement

This study was approved by the Institutional Review Board of CHA Gangnam Medical Center (IRB number: 09–06), and written informed consent was obtained from all participants.

**Figure 1:**
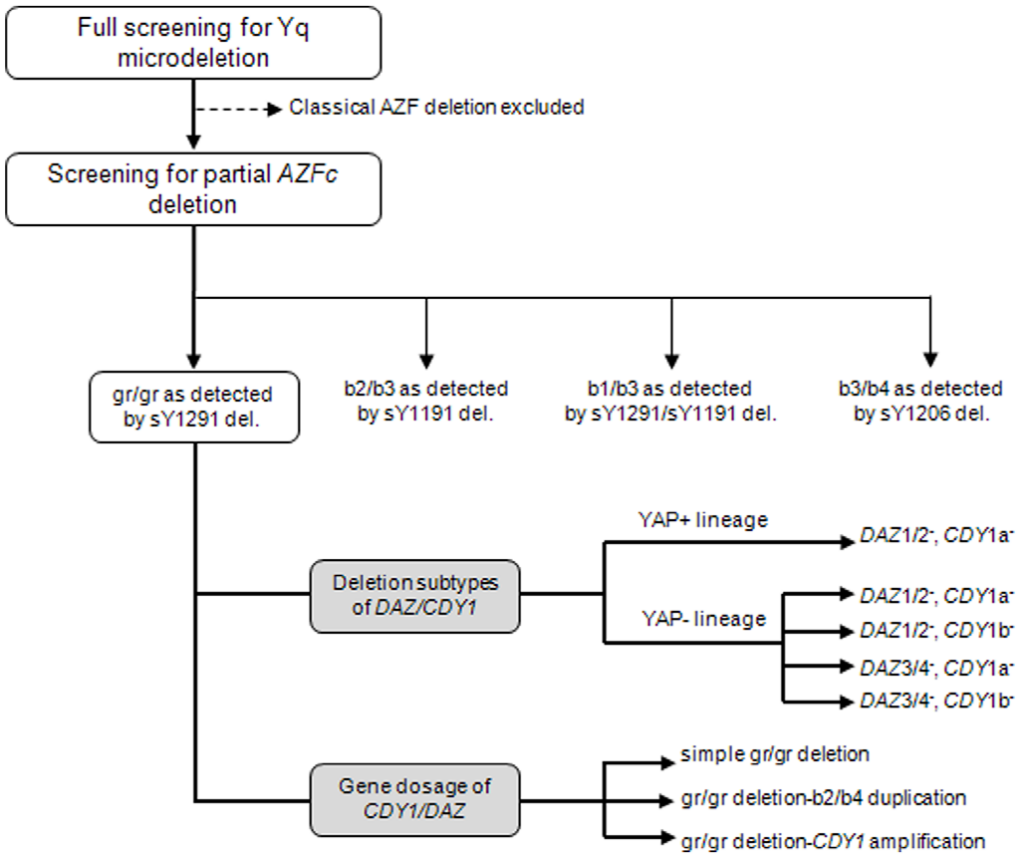
Analysis scheme.

**Table 1:** The distribution of partial AZFc deletion in groups with different spermatogenic status.

Partial deletion type	Azoo-/oligozoospermia (n = 377)	Normozoospermia (n = 217)	*P-*values	OR	95% CI
gr/gr deletion	32 (8.5%)	5 (2.3%)	**0.003**	3.933	1.509–10.250
b2/b3 deletion	22 (5.8%)	9 (4.1%)	0.373	1.432	0.647–3.169
b1/b3 deletion	4 (1.1%)	0	0.302	0.989	0.979–1.000
b3/b4 deletion	1 (0.3%)	0	1.000	0.997	0.992–1.003
Total	59 (15.6%)	14 (6.5%)	**0.001**	2.690	1.388–4.239

Compared between the groups with azoo-/oligozoospermia and normozoospermia, significant of *P*<0.05 are marked in bold.

### Study Population

A total of 619 men who were born to ethnic Korean parents, were analyzed for classical *AZF* deletions and partial *AZF*c region deletions in the Fertility Center of CHA Gangnam Medical Center between January 2009 and December 2010. Of these, 210 patients were excluded because they had either numerical or structural chromosome abnormalities, known causes of spermatogenic failure (such as obstruction of the vas deferens, history of orchitis and active orchitis, or history of unilateral, bilateral cryptochidism and varicocele) or insufficient clinical data. In addition, 32 patients with classical *AZF* deletions were also excluded. Thus, the subjects were composed of 377 men with azoospermia or oligozoospermia (sperm concentration of <20×10^6^/ml, in all three semen analyses). The normozoospermic control group comprised 217 men who consulted the same hospital for a routine fertility work-up. All of the control subjects were clinically healthy and possessed sperm concentrations of >20×10^6^/ml, normal sperm motility and morphology, and hormonal parameters. Semen analysis was performed according to the World Health Organization criteria [23].

**Figure 2:**
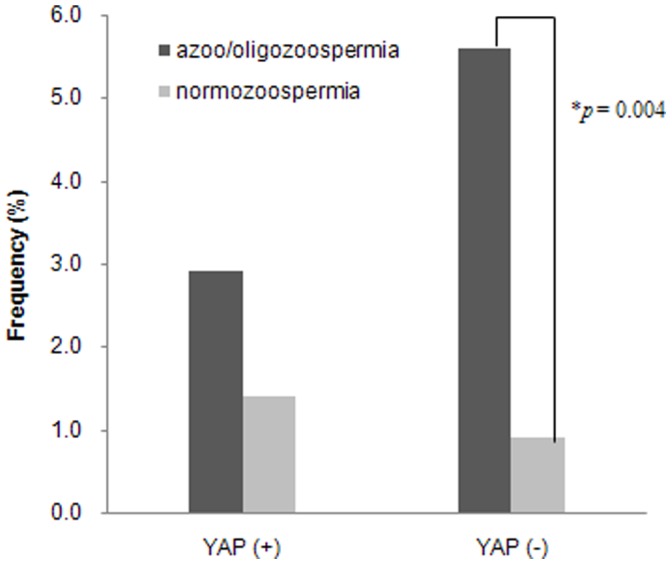
Comparison of gr/gr deletion frequencies between azoo-/oligozoospermic and normozoospermic groups in YAP+ and YAP− haplogroups. * Fisher’s exact test (two-tailed), OR = 6.341 (95% CI = 1.472–27.312), significant at *P*<0.05.

### Characterization of the Partial *AZF*c Deletions

Genomic DNAs were extracted from peripheral blood samples using the QIAamp® DNA Blood Mini Kit (QIAGEN, Hilden, Germany). Multiplex PCR reactions were performed using three STSs for the *AZF*c region (sY1191, sY1291, sY1206) and organized into two multiplex PCRs including a PCR control marker (*SOHLH2*). The amplification conditions were 94°C for 5 min, and then 30 cycles of 95°C for 30 sec, 61°C for 30 sec, and 72°C for 30 sec and a final elongation at 72°C for 10 min. The PCR reaction was always performed with a male control sample, a female sample, and a blank sample. The reaction products were analyzed by electrophoresis on 2% agarose gel. We identified partial *AZF*c deletions by the following STS results: the absence of sY1191, sY1291, sY1191/sY1291, and sY1206 represents the b2/b3, gr/gr, b1/b3, and b3/b4 deletions, respectively [7], [19].

**Table 2:** The frequency of gr/gr deletion subtypes by gene copy types of *DAZ*-*CDY1*, divided on the basis of their Y-haplogroup.

Group	YAP+ (n = 14)		YAP− (n = 580)	
	Azoo-/oligozoospermia(n = 11)	Normozoospermia(n = 3)	Azoo-/oligozoospermia(n = 366)	Normozoospermia(n = 214)
*DAZ*1/*DAZ*2^−^, *CDY*1a^−^	11(100%)	3 (100%)	5 (1.4%)	0
*DAZ*1/*DAZ*2^−^,*CDY*1b^−^	0	0	5 (1.4%)	1 (0.5%)
*DAZ*3/*DAZ*4^−^,*CDY*1a^−^	0	0	3 (0.8%)	0
*DAZ*3/*DAZ*4^−^, *CDY*1b^−^	0	0	8 (2.2%)	1 (0.5%)

Compared between the groups with azoo-/oligozoospermia and normozoospermia, no significant difference by fisher’s exact test (two-tailed), *P*>0.05.

### YAP Haplotype Analysis

Two haplogroups, YAP+ lineage (hgr.DE) and YAP− lineage [hgr. Y*(xDE)], were analyzed in samples with gr/gr deletion. Insertion/deletion polymorphisms of the YAP element was detected by PCR amplification using flanking primers as described in Hammer and Horai [24].

**Figure 3:**
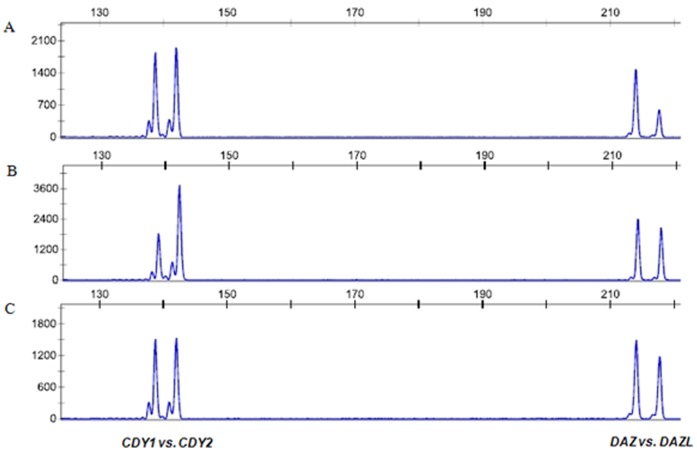
Examples of electrophoretograms showing different gene dosages of *CDY*1/*CDY*2 and *DAZ*/*DAZL* genes. The *x*-axis shows length of PCR products in base pairs and y-axis shows fluorescent intensity. The gene dosage of *CDY*1 and *DAZ* can be calculated by the comparison of peak area with *CDY*2 and *DAZL*, respectively, as internal standard with known number of copies. (A) Gene dosage of *CDY*1 and *DAZ* showed the 1∶1 of *CDY*1/*CDY*2 and 2∶1 of *DAZ*/*DAZL* patterns (in cases of no deletion according to the reference sequence or gr/gr deletion-b2/b4 duplication) (B) Gene dosage of *CDY*1 and *DAZ* identified by the 0.5∶1 of *CDY*1/*CDY*2 and 1∶1 of *DAZ*/*DAZL* patterns (in case of gr/gr deletion) (C) Gene dosage of *CDY*1 and *DAZ* identified by the 1∶1 of *CDY*1/*CDY*2 and 1∶1 of *DAZ*/*DAZL* patterns (in case of gr/gr deletion-*CDY*1 amplification).

**Table 3:** The frequency of gr/gr rearrangements according to analyses of *DAZ*-*CDY*1 gene copy number.

Rearrangement type	Azoo-/oligozoospermia (n = 377)	Normozoospermia (n = 217)	*P-*values	OR	95% CI
gr/gr del	26 (6.9%)	5 (2.3%)	**0.015**	3.141	1.188–8.303
gr/gr del-b2/b4 dupl	5 (1.3%)	0	0.164	0.987	0.975–0.998
gr/gr del-*CDY*1 ampl	1 (0.3%)	0	1.000	0.997	0.992–1.003

Compared between the groups with azoo-/oligozoospermia and normozoospermia, significant of P<0.05 are marked in bold.

### gr/gr Subtypes Analysis

Deletion copy types of *CDY*1 and *DAZ* gene were analyzed by the previous described method [14]. For *DAZ*, a sequence family variant (SFV), STS-sY587 placed in intron 10, which discriminates *DAZ*1/2 from *DAZ*3/4, was used. And for *CDY*1, we used a C/A SFV located in 7750 bp upstream from the *CDY*1 translation start codon (*CDY1-*7750), which distinguishes *CDY*1a from *CDY*1b. SFVs were analyzed by PCR followed by restriction enzyme digestion: *DAZ*-sY587/DraI (*DAZ*1/2 cut); *CDY*1-7750/PvuII (*CDY*1b cut). For quantitative analysis of *CDY*1 and *DAZ*, quantitative fluorescent-PCR (QF-PCR) was performed according to the previously described method [14], [25]. Briefly, *CDY*1 and *DAZ* were co-amplified with *CDY*2 and *DAZL*, respectively as a control with known gene copies. One primer (forward primer) in each set was labeled with 6-FAM (fluorescein amidite) fluorescent dye. The amplified products were loaded on the ABI 3130xl genetic analyzer and analyzed with the GeneMapper ID®version 3.2 (Applied Biosystems, Foster City, CA). The copy numbers of the genes were estimated based on the relative *DAZ/DAZL* and *CDY*1/*CDY*2 ratios.

### Statistical Analysis

Statistical analysis was performed using the statistical package SPSS for Windows (version 20, Chicago, IL, USA) software. The frequency of azoo-/oligozoospermia compared with normozoospermia was analyzed using both the chi-square test and Fisher’s exact test (two-tailed). Continuous variables were analyzed by the *t-* test for independent samples. Probability (*p*) values <0.05 were considered statistically significant.

## Results

A total of 377 azoo-/oligozoospermic patients and 217normozoospermic men were analyzed by this combined method described in Figure 1. Four types of partial *AZFc* deletions, the gr/gr, b2/b3, b1/b3, and b3/b4 deletions, were identified in this study (Table 1). Partial *AZFc* deletions were more frequently found in men with spermatogenic impairment than in the control group [59/377, 15.6% vs. 14/217, 6.5%, *p* = 0.001; odds ratio (OR) = 2.690; 95% confidence interval(CI) = 1.388–4.239]. Among them, the frequencies of gr/gr deletions were significantly higher in men with azoo-/oligozoospermia than normozoospermic men (32/377, 8.5% vs. 5/217, 2.3%, *p* = 0.003; OR = 3.933; 95% CI = 1.509–10.250), while the frequency of the b2/b3 deletion did not differ between men with azoo-/oligozoospermia and normozoospermia (22/377, 5.8% vs. 9/217, 4.1%). The b1/b3 (4/377, 1.1%) and b3/b4 (1/377, 0.3%) deletions were observed in the azoo-/oligozoospermic groups, but not in the normozoospermic controls.

As shown in Figure 2, men with gr/gr deletion were divided into two subgroups based on Y-haplogroups, YAP+ (14cases) and YAP− (23 cases). The frequency of men with gr/gr deletion/YAP+ haplogroup was the similar distributions in both groups; azoo-/oligozoospermia (11/377, 2.9%) and normozoospermic group (3/217, 1.4%). While the frequency of men with gr/gr deletion/YAP− haplogroup was significantly higher in the azoo-/oligozoospermic men than in the normozoospermic men (21/377, 5.6% vs. 2/217, 0.9%, *p* = 0.004; OR = 6.341; 95% CI = 1.472–27.312).

For further characterization of gr/gr deletions, we classified gr/gr deletions into four additional subtypes based on the deletion types of *CDY1* and *DAZ* gene copies (Table 2). The YAP+ lineage carried only one deletion subtype, *DAZ*1/2^−^, *CDY*1a^−^, with similar frequency in both azoo-/oligozoospermic and normozoospermic groups. On the other hand, in YAP**−** lineage, there were four deletion subtypes, *DAZ*1/2^−^, *CDY*1a^−^; *DAZ*1/2^−^, *CDY1b* ^−^; *DAZ*3/4^−^, *CDY*1a^−^ and *DAZ*3/4^−^, *CDY*1b^−^. Two types, *DAZ*1/2^−^, *CDY*1a^−^ and *DAZ*3/4^−^, *CDY*1a^−^, were found in only spermatogenetic impairment group.

Quantitative analysis of *CDY*1 and *DAZ* showed three gr/gr rearrangements, simple gr/gr deletion, gr/gr deletion-b2/b4 duplication, and gr/gr deletion-*CDY1* amplifications (Fig. 3). The frequency of the simple gr/gr deletion, which is the presence of one *CDY1* and two *DAZ* copies, were significantly different between azoo-/oligozoospermic (26/377, 6.9%) and normozoospermic (5/217, 2.3%) (*p* = 0.015, OR = 3.141; 95% CI = 1.188–8.303). Whereas, the gr/gr deletion-b2/b4 duplication (gr/gr deletion followed by b2/b4 duplication with the presence of two *CDY1* and four *DAZ* copies) and gr/gr deletion-*CDY*1 amplication (gr/gr deletion with the presence of two *CDY1* and two *DAZ* copies) were found in only azoo-/oligozoospermic group with frequencies of 1.3% (5/377) and 0.3% (1/377), respectively (Table 3). We also compared the mean total sperm concentration in azoo-/oligozoospermic groups. There was no significant difference between the subjects with simple gr/gr deletion and gr/gr deletion-b2/b4 duplication (5.6±15.8×10^6^/ml vs. 5.0±11.2×10^6^/ml, respectively).

## Discussion

In this study, we investigated the types of partial *AZF*c deletions and their clinical implications in Korean population. Firstly, we screened Yq microdeletions using STS markers for *AZF*a, *AZF*b and *AZF*c region. And then, gr/gr deletions were classified according to the Y-haplogroup, deletion copy types and gene copy number of *CDY*1 and *DAZ* genes.

Regardless of deletion type, as we expected, the overall frequency of partial *AZF*c deletions in azoo-/oligozoospermic men was higher than in normozoospermic men (15.6% vs. 6.5%, *p* = 0.001). This result suggested that such mutations could be a risk factor for impaired spermatogenesis in the Korean population. Four types of partial *AZF*c deletions were identified in our population and only gr/gr deletions were statistically associated with impaired spermatogenesis (Table 1). Our result is consistent with two recent meta-analyses [20], [26] and with the largest study on Caucasians [21]. However, several other studies showed no association between gr/gr deletions and azoo-/oligozoospermia in Caucasian [10], [27], Asian [4], [28]–[31] and admixed ethnic populations [32]. The phenotype of gr/gr deletion carriers is reported in Table S1. For the other three types, the frequency of b2/b3deletion was not significant difference in between azoo-/oligozoospermic males and normozoospermic controls, which suggested that b2/b3 deletion is not associated with spermatogenetic impairment in our study population. Similar observations have been reported in various studies [6], [19], [27], [33]. Data from very large study populations from China and, very recently, a North African population suggest that b2/b3 deletion is a risk factor for impaired sperm production [28], [29], [34]. However, the limited number of subjects with this deletion in our study does not allow to define its role in the Korean population. The b1/b3 and b3/b4 deletions were identified only in patients with azoo-/oligozoospermia indicating that deletions might affect on spermatogenesis with mechanism yet to be revealed. The results, however, were limited due to the small number of cases. We compared the clinical features including total sperm count, combined testicular volume, FSH and testosterone levels between subjects with gr/gr deletion and others (Table S2). No statistically significant differences in sperm count were found when comparing sperm count of gr/gr deletion carriers versus non carriers. This may derive from the peculiar composition of our study population which shows a high prevalence of azoospermic men. Gr/gr deletion carriers are mainly oligozoospermic and in fact this deletion is more likely to be associated with oligozoospermia than with azoospermia [21], [26].

The overall frequency of gr/gr deletions in Korean patient with spermatogenetic failure (8.5%) was higher than Europeans (∼4.5%) and similar to Han Chinese populations (10.0 and 10.6%) [11], [26]. This might be resulting from different origins of the study populations. It has been reported that some Asian populations, including Korean, Japanese, and Tibetan, showed the higher frequency of YAP+ haplogroup compared to other populations [35], [36]. And the haplogroup D2b derived from YAP+ lineage always possessed gr/gr deletion and showed normal phenotype [31], [37]. So, we reclassified gr/gr deletions based on YAP haplogroups, YAP+ (hgr.DE) and YAP− [hgr.Y*(xDE)] lineages. Our data showed that the frequency of gr/gr deletion with YAP+ lineage was not significantly different between azoo-/oligozoospermic males and normozoospermic controls. However, the frequency of gr/gr deletion with YAP− lineage was much higher in azoo-/oligozoospermic males than in controls (Fig. 2). So, we concluded that only gr/gr deletion with YAP**−** lineage was associated with spermatogenetic impairment in Korean population. Hereby, we demonstrated that the gr/gr deletion might effect on spermatogenetic impairment inY haplogroup-dependent manner.

We also investigated gr/gr deletion subtypes according to deletion patterns of *DAZ* and *CDY1* gene copies. Normally, four copies of *DAZ* gene and two copies of *CDY1* gene are assigned in the *AZF*c region. Several studies related to gr/gr deletion subtypes and spermatogenetic impairments have presented different conclusions. Some studies showed *DAZ*1/2 deletions were associated with spermatogenetic impairment [17], [19], [21], [38], [39], and Machev et al. [14] presented *DAZ*3/4-*CDY*1a deletions were linked to the phenotype. More recently, no association between subtypes of gr/gr deletion and phenotypic abnormalities was also reported [6]. In our study, subjects with YAP+ lineage showed a homogeneous pattern of gr/gr deletion, *DAZ*1/2^−^, *CDY*1a^−^ and no significant difference between azoo-/oligozoospermic and normozoospermic controls. This result was the same as a previous report [37]. In YAP− lineage, deletion of the *CDY*1a was found only in spermatogenetic impairment group (Table 2). Although our result might not be sufficient to verify the association between these deletion subtypes and clinical consequences, this phenomenon is similar to previous studies in a correlation between the absence of *CDY*1a and male infertility [14], [39]. So, further studies will be required.

Finally, we carried out *CDY*1 and *DAZ* gene copy number analysis to identify gr/gr rearrangement types (Table 3). Krausz et al. [6] reported that gr/gr deletions could be classified into five rearrangement types based on the copy number of *CDY*1 and *DAZ* gene. In our study, three out of five rearrangement types were identified and the majority of gr/gr deletions (83.8%, 31/37) were simple gr/gr deletion type, one copy of *CDY*1 and two copies of *DAZ*. This result was similar to European population (80%, 128/160) [6]. The other two types, gr/gr deletion-b2/b4 duplications (four copies of *DAZ* and two copies of *CDY*1) and gr/gr deletion-*CDY*1 amplification (two copies of *DAZ* and two copies of *CDY*1), were found in patients with spermatogenetic impairment with frequencies of 13.5% (5/37) and 2.7% (1/37), respectively, but none in normozoospermic controls. These results suggested that regardless of having an additional duplication of *AZF*c region, the simple gr/gr deletion might be associated with spermatogenetic impairment in Korean population. Recently, Lu et al. [22] reported that b2/b3 deletion with secondary duplication was also a risk factor for spermatogenetic impairment in Han Chinese population. Meanwhile, two possible mechanisms to generate the gr/gr deletion-*CDY1* amplification are proposed and the recombinant products resulting from both ways were not distinguishable as shown in Figure S1. So, we could not verify what happened first with current technology.

In conclusion, we analyzed 377patients with spermatogenetic impairments and 217normozoospermic controls. As far as we know, this is the first report that only gr/gr deletion with YAP− lineage, among several types of partial *AZF*c deletions, was associated with spermatogenetic impairment in Korean population. Although the role of *CDY*1 and *DAZ* on spermatogenesis is still not clear, further studies on other genes related to spermatogenesis and larger scale population study will be assured to understand the spermatogenesis pathology.

## Supporting Information

Figure S1 **Two possible mechanisms of gr/gr del-** ***CDY1*** **amplification;** one is that the g1/g2 recombination resulting in gr/gr deletion arises first and then the *CDY1* amplification occurs and the other is vice versa. The recombinant products from both ways are not distinguishable.
(TIF)

Table S1 **Clinical features of patients bearing gr/gr deletions and their deletion patterns based on the type and number of** ***DAZ*** **-** ***CDY*** **1copies deleted.**
(DOCX)

Table S2 **Means±SD of the total sperm count, testicular volume and hormonal levels in patients and in controls.**
(DOCX)
