# Supplementary figures and images for: Impaired Spermatogenesis and gr/gr Deletions Related to Y Chromosome Haplogroups in Korean Men
Source: PLoS One. 2012 Aug 23;7(8):e43550. doi: 10.1371/journal.pone.0043550 (PMC3426531; doi:10.1371/journal.pone.0043550)

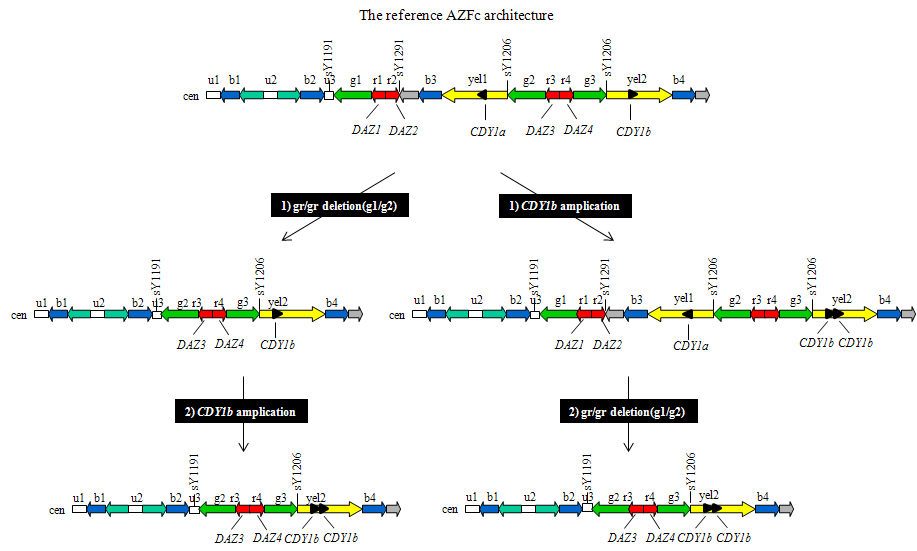

Supplement: Figure S1 — Two possible mechanisms of gr/gr del- CDY1 amplification; one is that the g1/g2 recombination resulting in gr/gr deletion arises first and then the CDY1 amplification occurs and the other is vice versa. The recombinant products from both ways are not distinguishable. (TIF) [file pone.0043550.s001.tif]
