# Supplementary material for: Impaired Spermatogenesis and gr/gr Deletions Related to Y Chromosome Haplogroups in Korean Men
Source: PLoS One. 2012 Aug 23;7(8):e43550. doi: 10.1371/journal.pone.0043550 (PMC3426531; doi:10.1371/journal.pone.0043550)
Supplement: Table S1 — Clinical features of patients bearing gr/gr deletions and their deletion patterns based on the type and number of DAZ - CDY 1copies deleted. (DOCX) [file pone.0043550.s002.docx]

Table S1. Clinical features of patients bearing gr/gr deletions and their deletion patterns based on the type and number of *DAZ*-*CDY*1copies deleted.

| Case no. | Total sperm concentration ( X 106/ml) | Combined  testicular  volume (mL) | FSH (mlU/mL) | Y hgr | Deletion subtype | Rearrangement type |
| --- | --- | --- | --- | --- | --- | --- |
| 4917 | 0 | 16 | 20.4 | − | *DAZ*1/2^-^, *CDY*1a^-^ | gr/gr del |
| 4927 | 0 | 11 | 32.3 | − | *DAZ*3/4^-^, *CDY*1a^-^ | gr/gr del |
| 4999 | 0 | 16 | 38.2 | − | *DAZ*3/4^-^, *CDY*1b^-^ | gr/gr del |
| 5017 | 0 | 10 | 21.9 | + | *DAZ*1/2^-^, *CDY*1a^-^ | gr/gr del |
| 5039 | 0 | 32 | 22.1 | + | *DAZ*1/2^-^, *CDY*1a^-^ | gr/gr del |
| 5053 | 0 | 20 | 14.3 | − | *DAZ*1/2^-^, *CDY*1b^-^ | gr/gr del |
| 5076 | 0 | 10 | 30.7 | + | *DAZ*1/2^-^, *CDY*1a^-^ | gr/gr del |
| 5080 | 0 | 24 | 16.2 | − | *DAZ*1/2^-^, *CDY*1a^-^ | gr/gr del |
| 5121 | 0 | 12 | 36.3 | + | *DAZ*1/2^-^, *CDY*1a^-^ | gr/gr del |
| 5132 | 0 | 11 | 33.2 | + | *DAZ*1/2^-^, *CDY*1a^-^ | gr/gr del |
| 5134 | 0 | 36 | 24.6 | − | *DAZ*3/4^-^, *CDY*1b^-^ | gr/gr del-b2/b4 dupl |
| 5140 | 0 | 14 | 56.5 | + | *DAZ*1/2^-^, *CDY*1a^-^ | gr/gr del-b2/b4 dupl |
| 5186 | 0 | 26 | 29.8 | + | *DAZ*1/2^-^, *CDY*1a^-^ | gr/gr del-b2/b4 dupl |
| 5338 | 0 | 28 | 36.9 | − | *DAZ*3/4^-^, *CDY*1b^-^ | gr/gr del |
| 5339 | 0 | 50 | 2.3 | − | *DAZ*3/4^-^, *CDY*1b^-^ | gr/gr del |
| 5343 | 0 | 20 | 25.9 | − | *DAZ*1/2^-^, *CDY*1b^-^ | gr/gr del |
| 5376 | 0 | 2 | 0.3 | − | *DAZ*3/4^-^, *CDY*1b^-^ | gr/gr del |
| 5335 | <0.01 | 16 | 38.3 | + | *DAZ*1/2^-^, *CDY*1a^-^ | gr/gr del |
| 4975 | <0.01 | 30 | 5.8 | + | *DAZ*1/2^-^, *CDY*1a^-^ | gr/gr del |
| 5106 | <0.01 | 18 | 15.3 | − | *DAZ*3/4^-^, *CDY*1b^-^ | gr/gr del |
| 5694 | 0.1 | 36 | 4.7 | + | *DAZ*1/2^-^, *CDY*1a^-^ | gr/gr del |
| 5083 | 0.2 | 28 | 19.0 | − | *DAZ*1/2^-^, *CDY*1b^-^ | gr/gr del |
| 5045 | 0.3 | 14 | 10.3 | − | *DAZ*1/2^-^, *CDY*1a^-^ | gr/gr del |
| 5110 | 0.3 | 32 | 26.2 | − | *DAZ*1/2^-^, *CDY*1b^-^ | gr/gr del-CDY1 ampl |
| 5092 | 2.6 | 20 | 17.4 | − | *DAZ*1/2^-^, *CDY*1a^-^ | gr/gr del |
| 5149 | 4.0 | 40 | 6.7 | + | *DAZ*1/2^-^, *CDY*1a^-^ | gr/gr del-b2/b4 dupl |
| 4918 | 8.0 | 28 | 7.5 | − | *DAZ*3/4^-^, *CDY*1b^-^ | gr/gr del-b2/b4 dupl |
| 5272 | 12.7 | 36 | 8.9 | − | *DAZ*3/4^-^, *CDY*1b^-^ | gr/gr del |
| 5755 | 14.6 | 32 | 7.6 | − | *DAZ*1/2^-^, *CDY*1b^-^ | gr/gr del |
| 5344 | 25.0 | 36 | 10.2 | − | *DAZ*3/4^-^, *CDY*1a^-^ | gr/gr del |
| 5284 | 27.2 | 36 | 4.0 | − | *DAZ*1/2^-^, *CDY*1a^-^ | gr/gr del |
| 4937 | 76.5 | 30 | 7.0 | − | *DAZ*3/4^-^, *CDY*1a^-^ | gr/gr del |
