# Supplementary material for: Impaired Spermatogenesis and gr/gr Deletions Related to Y Chromosome Haplogroups in Korean Men
Source: PLoS One. 2012 Aug 23;7(8):e43550. doi: 10.1371/journal.pone.0043550 (PMC3426531; doi:10.1371/journal.pone.0043550)
Supplement: Table S2 — Means±SD of the total sperm count, testicular volume and hormonal levels in patients and in controls. (DOCX) [file pone.0043550.s003.docx]

Table S2. Means ± SD of the total sperm count, testicular volume and hormonal levels in patients and in controls

| Group |  | Total sperm count ( X 10^6^/ml) | Combined  testicular volume (mL) | FSH (mlU/mL) | Testosterone (ng/mL) |
| --- | --- | --- | --- | --- | --- |
| Patients | YAP+ (n=11)^a^ | 0.4 ± 1.2* | 21.5± 11.4 | 26.0 ± 16.0 | 3.1 ± 1.4 |
|  | YAP- (n=366)^b^ | 3.2 ± 8.9 | 23.7 ± 9.7 | 19.5 ± 13.4 | 3.6 ± 1.3 |
|  |  |  |  |  |  |
|  | with gr/gr deletion (n=32)^a^ | 5.4 ± 14.8 | 24.1 ± 11.1 | 19.7 ± 13.5 | 3.7 ± 1.6 |
|  | with b2/b3 deletion (n=22)^b^ | 1.9 ± 7.5 | 21.8 ± 10.4 | 18.0 ± 14.5 | 3.4 ± 1.3 |
|  | with partial *AZFc* deletion (n=59)^b^ | 4.2 ± 12.5 | 22.8 ± 10.8 | 18.7 ± 13.2 | 3.7 ± 1.6 |
|  | without partial *AZFc* deletion (n=318)^b^ | 2.9 ± 7.9 | 23.8 ± 9.5 | 19.9 ± 13.5 | 3.6 ± 1.3 |
|  |  |  |  |  |  |
| Control | YAP+ (n=3)^a^ | 248.6 ± 164.9 | 50.0 ± 0 | 3.4 ± 0.5 | 4.9 ± 1.8 |
|  | YAP- (n=214)^b^ | 225.4 ± 195.1 | 39.9 ± 9.5 | 5.0 ± 2.2 | 4.4 ± 1.9 |
|  |  |  |  |  |  |
|  | with gr/gr deletion (n=5)^a^ | 311.2 ± 145.5 | 47.5 ± 5.0 | 4.0 ± 1.1 | 4.5 ± 1.4 |
|  | with b2/b3 deletion (n=9)^b^ | 209.3 ± 91.5 | 41.9 ± 20.5 | 4.3 ± 2.0 | 4.3 ± 1.0 |
|  | with partial *AZFc* deletion (n=14)^b^ | 245.7 ± 119.3 | 44.0 ± 8.9 | 4.2 ± 1.7 | 4.3 ± 1.1 |
|  | without partial *AZFc* deletion (n=203)^b^ | 224.3 ± 199.0 | 39.8 ± 9.5 | 5.1 ± 2.3 | 4.4 ± 1.9 |

The differences were compared between a and b.

* Significant difference by Student's t test (two-tailed) P<.0001
